# FINANCIAL EXPLOITATION PREVENTION AMONG OLDER ADULTS WITH DEMENTIA IN RURAL MICHIGAN: A PILOT RANDOM CONTROL TRIAL

**DOI:** 10.1093/geroni/igae098.1400

**Published:** 2024-12-31

**Authors:** Fei Sun, Yu Guo, Zhenmei Zhang, Xia Wang, Haneul Kim, Brittany Tucker, Eleanor Smith

**Affiliations:** Michigan State University, Haslett, Michigan, United States; Xi’an Jiaotong University, Xi’an, Shaanxi, China (People’s Republic); Michigan State University East Lansing, East Lansing, Michigan, United States; Arizona State University, Phoenix, Arizona, United States; Michigan State University East Lansing, East Lansing, Michigan, United States; Michigan State University East Lansing, East Lansing, Michigan, United States; Michigan State University East Lansing, East Lansing, Michigan, United States

## Abstract

Dementia increases the susceptibility of older adults to financial exploitation, including abuse by relatives or acquaintances and fraud by strangers. This study evaluated the effectiveness of a novel community-based financial exploitation prevention model for older adults with dementia residing in rural Michigan. Drawing from the routine activity theory, the study focused on mitigating financial exploitation risks linked to family caregivers. While family caregivers might potentially engage in such exploitation, they also play a crucial role in detecting and preventing fraudulent activities. Employing a randomized control design, 32 family caregivers in intervention groups and 23 in control groups received an education session on dementia, financial exploitation, and community resources. In addition, intervention groups received 6 sessions of case management service tailored to mitigate identified risk factors for financial exploitation. Linear mixed modeling (LMM) was applied to assess changes before and post intervention and between groups on the following outcomes: attitude towards financial exploitation, competence in addressing financial exploitation, caregiver stress, and dementia knowledge and stigma. No significant differences were found on dementia knowledge and attitude towards financial exploitation. However, caregivers in the intervention groups exhibited significantly greater confidence in addressing financial exploitation and reported lower levels of dementia-related stigma (p <.05) compared to the control group. Both groups reported a decrease in caregiver stress, but no group difference was observed. The study highlights the integral role of family caregivers as a protective shield against financial exploitation, and the need for policy and practice efforts to educate and support family caregivers.

